# GLP-1/Exendin-4 induces β-cell proliferation via the epidermal growth factor receptor

**DOI:** 10.1038/s41598-017-09898-4

**Published:** 2017-08-22

**Authors:** Joseph Fusco, Xiangwei Xiao, Krishna Prasadan, Qingfeng Sheng, Congde Chen, Yung-Ching Ming, George Gittes

**Affiliations:** 1 Children’s Hospital of Pittsburgh of the University of Pittsburgh Medical Center, Department of Pediatric Surgery, Pittsburgh, PA 15224 USA; 2 Shanghai Children’s Hospital, Shanghai Jiao Tong University, Department of General Surgery, Minhang Qu, 200240 China; 3 The 2nd Affiliated Hospital & Yuying Children’s Hospital of Wenzhou Medical University, Wenzhou, Zhejiang, 3250527 China; 4 Chang Gung Memorial Hospital, Chang Gung University, Department of Pediatric Surgery, Taipei City, 105 Taiwan

## Abstract

Exendin-4 is a long acting glucagon-like peptide 1 (GLP-1) analogue that is an agonist for the GLP-1 receptor, a G-protein coupled receptor (GPCR). Exendin-4 is used to clinically improve glucose tolerance in diabetic patients due to its ability to enhance insulin secretion. In rodents, and possibly in humans, exendin-4 can stimulate β-cell proliferation. The exact mechanism of action to induce β-cell proliferation is not well understood. Here, using a β-cell specific epidermal growth factor receptor (EGFR) null mouse, we show that exendin-4 induced an increase in proliferation and β-cell mass through EGFR. Thus, our study sheds light on the role of EGFR signaling in the effects of exendin-4 on the control of blood glucose metabolism and β-cell mass.

## Introduction

Inducing β-cell proliferation is an attractive treatment modality in the diabetic patient^1^. Exogenous insulin aids in glucose control, but is inherently different than endogenous insulin, which prevents the microvascular and macrovascular complications of diabetes. Recent studies have begun to elucidate the signaling pathways involved in β-cell proliferation, particularly in the mature β-cell^2^.

The epidermal growth factor receptor 1 (EGFR) is one of four erbB transmembrane tyrosine kinase receptors. Binding of ligand leads to dimerization and autophosphorylation of a tyrosine kinase, which in turn leads to activation of the epidermal growth factor (EGF) intracellular signaling pathway^3^. Within the pancreas, EGFR is predominantly expressed in islets, exerting its effects on β-cell development, function, and proliferation^4^. EGFR null mutant mice have delayed β-cell differentiation and impaired β-cell proliferation during organogenesis^5^. Additional evidence of this impairment was displayed with inhibition of EGFR postnatally^6^, in pregnancy^7^, and with high fat diet^7^. Recently, we found that EGFR was upregulated in islets after partial pancreatectomy. In the absence of β-cell EGFR, β-cell proliferation was reduced at baseline and after partial pancreatectomy^8^.

Glucagon-like peptide 1 (GLP-1) is an incretin hormone that is a potent β-cell mitogen, and also stimulates insulin synthesis and secretion. Exendin-4, a long-acting GLP-1 analogue, is resistant to DPP-IV cleavage and therefore more useful clinically^9^. It is not clear how GLP-1 signaling leads to the PI3-kinase activity seen in β-cell proliferation. Exendin-4 appears to activate the β-cell EGFR to promote β-cell proliferation^4, 7, 10, 11^. *In vitro*, it appears that GLP-1 or exendin-4 treatment leads to release of betacellulin and EGF ligands from the peri-islet extracellular matrix, with subsequent stimulation of β-cell proliferation^12^.

Here, we show that EGFR is necessary *in vivo* for these exendin-4-mediated effects. We employ a pancreatic β-cell specific EGFR null mouse (β-EGFR^fx/fx^) and exogenous exendin-4 treatment to show a role for β-cell EGFR in β-cell mass, islet size, β-cell proliferation, and insulin content.

## Materials and Methods

### Mouse Manipulations

The Animal Research and Care Committee at the Children’s Hospital of Pittsburgh and the University of Pittsburgh IACUC approved all mouse experiments. All experiments were performed in accordance with relevant guidelines and regulations. C57BL/6 mice were purchased from the Jackson Laboratory (Bar Harbor, ME, USA). Transgenic mice expressing EGFR^fx/fx^ have been previously described^13^. EGFR^fx/fx^ mice were crossed with Pdx1-CreERT mice, which also have been previously described, to get Pdx1-CreERT; EGFR^fx/fx^ mice^8, 14^. The CreERT-negative littermates of same age were used as controls, which showed no phenotypic difference from wild-type C57/BL6 mice. All of these mice have a C57/BL6 background. The terminology used henceforth to distinguish amongst groups is as follows: Pdx1-CreERT; EGFR^fx/fx^ without exendin treatment (Cre^+^Ex^−^), Pdx1-CreERT; EGFR^fx/fx^ with exendin treatment (Cre^+^Ex^+^), EGFR^fx/fx^ without exendin treatment (Cre^−^Ex^−^), and EGFR^fx/fx^ with exendin treatment (Cre^−^Ex^+^).

### Measurement of Glucose Tolerance, β-cell Mass, and Insulin Content

Glucose tolerance and β-cell mass were measured as previously described^1, 15^. Pancreatic samples were cut on the longitudinal axis allowing for calculation of β-cell mass as well as insulin content. Insulin content was determined from homogenate pancreatic tissue samples normalized to weight with the Mouse Insulin ELISA Kit (MyBioSource Inc, San Diego, CA, USA).

### Tamoxifen and Exendin-4 Injection

Tamoxifen (Sigma-Aldrich, St. Louis, MO, USA) was dissolved in 10 mg/ml corn oil (Sigma-Aldrich, St. Louis, MO, USA) and 1 mg/20 g body weight per day of intraperitoneal tamoxifen was given to 6-week-old mice for 5 consecutive days in order to induce Cre recombination. Control mice also received tamoxifen at the same dose and frequency. Intraperitoneal injection of exendin-4 (Sigma-Aldrich, St. Louis, MO, USA) was performed 2 weeks after tamoxifen injection. Exendin-4 (0.1 μg/mg of body weight) was administered twice daily, while saline was administered to control mice.

### BrdU Incorporation, Quantification of Proliferating β-Cells

Mice were given BrdU drinking water for 1 week after they received 2 days of exendin-4 injection or saline injection, as described before^15, 16^. BrdU water was replaced every 3 days. Quantification of β-cell proliferation was based on the percentage of BrdU^+^ β-cells. At least 2,000 β-cells were counted for each mouse. If the percentage of BrdU^+^ or β-cells was low, more cells were counted until at least 50 BrdU^+^ β cells or 20 Ki-67^+^ β-cells were counted^15^.

### Pancreatic Digestion and Islet Isolation

Pancreas digestion and islet isolation procedures were performed as described before^17–19^. The pancreas was infused with 0.25 mg/ml collagenase (Sigma-Aldrich) for 40 min to obtain a single cell population. Islets were handpicked at least three times to avoid contamination from non-islet fractions. Total RNA was extracted from isolated islets and analyzed by rt-PCR to confirm the purity of the islets by the absence of amylase and CK19 at the transcriptional level.

### Western Blot Analysis

Western blotting analysis was performed as previously described (23, 38). Primary antibodies for Western blotting analysis were rabbit polyclonal anti-GAPDH (Cell Signaling, San Jose, CA) and rabbit anti-EGFR (Santa Cruz Biotechnology). Secondary antibody is HRP-conjugated anti-rabbit (Dako, Carpinteria, CA).

### Isolation of RNA and RT-qPCR

Total RNA was extracted using the RNeasy mini kit (Qiagen, Valencia, CA), and then quantified with the Nanodrop 1000 (Thermo Fisher Scientific, Inc., Waltham, MA), followed by cDNA synthesis (Qiagen) and RT-qPCR. RT-qPCR was done as described before^17–19^. The primers used were EGFR (QT00101584, Quiagen, Hilden, Germany), GLP1R (Fisher Scientific, Waltham, MA), and GAPDH (Fisher Scientific, Waltham, MA). Results were first normalized against the housekeeping gene GAPDH, which is stable across all samples, and then against the experimental controls.

### Glucose Stimulated Insulin Secretion (GSIS)

For *in vitro* static GSIS, digestion and islet isolation was performed as previously described^1, 15, 16^ for each condition. Mouse islets were cultured in Ham’s F10 medium (Life Technologies, St. Louis, MO, USA) supplemented with 0.5% BSA (Sigma-Aldrich, St. Louis, MO, USA), 2 mmol/L glutamine, 2 mmol/L calcium, and 5 mmol/L glucose at 37 °C, 95% air/5% CO_2_. After overnight culture, 200 islets per condition were transferred to new plates and treated with low glucose (2.8 mmol/L) and high glucose (16.7 mmol/L) conditions. Islets were pelleted by centrifugation and lysed in acid ethanol for assessment of insulin in media and islets by radioimmunoassay (Linco Research Inc., St. Charles, Missouri, USA). Results were reported as insulin secreted (ng/mL) per hour normalized to number of islets.

### Immunohistochemistry

Tissues were fixed in 4% paraformaldehyde overnight at 4 °C, followed by cryoprotection in 30% sucrose overnight, and finally snap-freezing. All samples were sectioned at 6 μm. For antigen retrieval, BrdU immunostaining slides were incubated in 2 mmol/liter HCl for 40 min. All slides were incubated with primary antibodies at 4 °C overnight, then incubated with secondary antibodies for 2 h at room temperature. Primary antibodies used in this study were: guinea pig polyclonal insulin (Dako), rabbit Ki-67 (Abcam), and rat BrdU (Abcam, Cambridge, MA). The secondary antibodies were: Cy2, Cy3, or Cy5-conjugated donkey streptavidin, anti-rabbit, anti-guinea pig, and anti-rat. Nuclear staining was performed with Hoechst staining (Becton-Dickinson Biosciences, San Jose, CA). Cryosection imaging was performed with the AxioImager Z.1 microscope (Zeiss).

### Data Analysis

GraphPad Prism 6.0 (GraphPad Software, Inc. La Jolla, CA) was used for statistical analyses. All values are depicted as mean ± S.E. Five repeats were analyzed in each condition. All data were statistically analyzed using one-way ANOVA with a Bonferroni correction, followed by Fisher’s Exact Test to compare two groups. Significance was considered when *p* < 0.05.

## Results

### Impaired glucose tolerance in a β-cell specific EGFR null mouse

**Figure 1:**
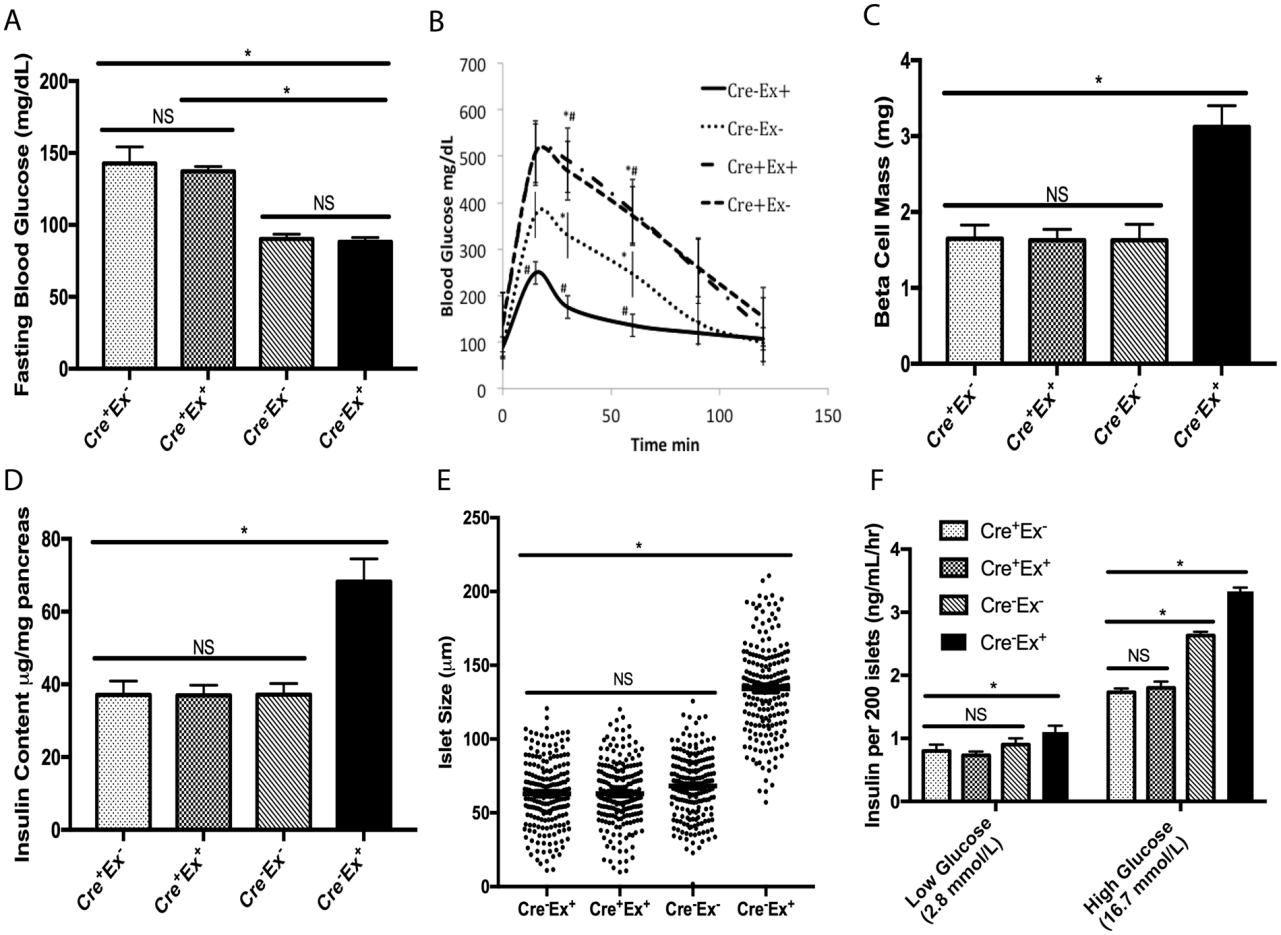
(**A**) Fasting glucose levels were increased in Cre^+^Ex^−^mice. This increase was not reversed by exendin-4 treatment (Cre^+^Ex^+^). (**B**) Glucose tolerance tests showed an enhanced ability to respond to a glucose load in control Cre^−^ mice with exendin-4 treatment (Cre^−^Ex^+^). A defect in glucose tolerance was seen in Cre^+^Ex^−^ mice that could not be salvaged with exendin-4 (Cre^+^Ex^−^). (**C**) β-cell mass was significantly increased in control mice as compared with Cre^+^ mice. (**D**) Insulin content mirrors β-cell mass. (**E**) Islet size was significantly increased in control mice after exendin-4 treatment (Cre^−^Ex^+^), as compared with Cre^+^Ex^+^ and Cre^+^Ex^−^ mice. n = 5. All results were mean ± S.E. * #, p < 0.05, NS, no significance). (**F**) *In vitro* GSIS shows significantly increased insulin release in control mice with exendin-4 treatment (Cre^−^Ex^+^) at low and high glucose concentrations. At high glucose concentrations, there is significantly less insulin secretion in Cre^+^ mice, regardless of treatment with exendin-4, as compared to control Cre^−^ mice.

Male pdx1-creERT;EGFR^fx/fx^ mice (Cre^+^) and control Cre^−^;EGFR^fx/fx^ mice received exendin-4 (Cre^+^Ex^+^ and Cre^−^Ex^+^) or saline injection (Cre^+^Ex^−^ and Cre^−^Ex^−^) for 5 days starting at 8 weeks of age, 2 weeks after tamoxifen treatment. There is loss of EGFR mRNA and protein expression after two weeks of tamoxifen treatment (Supplementary Fig. 1A,B). After 5 days of exendin-4 (Cre^+^Ex^+^ and Cre^−^Ex^+^) or saline (Cre^+^Ex^−^ and Cre^−^Ex^−^) intraperitoneal injection, glucose tolerance tests were performed to assess the ability of the mice to respond to a glucose load. Fasting glucose values were significantly higher in Cre^+^Ex^−^ mice compared to Cre^−^Ex^−^ controls at baseline (142 ± 11.4 mg/dL vs. 90 ± 3.3 mg/dL)(Fig. 1A). The fasting glucose levels when the Cre^+^ mice were treated with exendin-4 (Cre^+^Ex^+^) remained elevated (137.3 ± 3.3 mg/dL). However treatment of Cre^−^ mice with exendin-4 (Cre^−^Ex^+^) led to significantly lowered blood glucose values (Fig. 1B). The Cre^+^Ex^−^ mice displayed a decreased ability to respond to a glucose load, producing a diabetic phenotype (Fig. 1B). This diabetogenic effect was not rescued by the addition of exendin-4 (Cre^+^Ex^+^), suggesting that exendin-4 exerts its hypoglycemic effect through EGFR. These differences were significant at 15, 30, and 60 min (p < 0.05). These data suggest that EGFR is necessary for a normal insulin response, and that exendin-4 amplifies the insulin response through β-cell EGFR.

### Effect of EGFR on glucagon like peptide 1 receptor (GLP1-R)

As discussed above, there is loss of EGFR mRNA and protein with Cre recombination in the β-cell in this model. The recombination event has no effect on glucagon like peptide 1 receptor (GLP-1R) mRNA (Supplementary Fig. 1C). Therefore, there should be no differential effect on β-cell mass, islet size, β-cell proliferation, and insulin content with exendin-4 treatment due to GLP1-R expression.

### Effect of exendin-4 on β-cell mass

In accordance with previously published data^20^, β-cell mass was roughly doubled with exendin-4 treatment (Cre^−^Ex^+^, 3.12 ± 0.28 mg vs 1.63 ± 0.21 mg, p < 0.05)(Fig. 1C). Very large islets were seen throughout the pancreas after 5 days of exendin-4 injection (see below). However, the Cre^+^Ex^−^ mice showed no difference in β-cell mass from Cre^−^Ex^−^ controls at baseline (1.65 ± 0.18 mg). Interestingly, in Cre^+^Ex^+^mice, exendin-4 had no enhancing effect on β-cell mass (1.63 ± 0.14 mg, p < 0.05), further suggesting that EGFR is required for GLP-1 signaling to induce β-cell proliferation.

### Effect of exendin-4 on insulin content

The pancreas of Cre^−^ mice treated with exendin-4 (Cre^−^Ex^+^) had significantly higher insulin content than saline-treated Cre^−^Ex^−^ controls (68.2 ± 6.3 μg/mg of pancreas vs 37.2 ± 3.1 μg/mg of pancreas, p < 0.05) (Fig. 1D). This ratio corresponds well with the almost 2-fold increase in β-cell mass in Cre^−^Ex^+^ mice discussed above. This effect of exendin-4 is lost in the Cre^+^ mice (37.0 ± 2.8 μg/mg of pancreas, p < 0.05) supporting that EGFR is necessary for the insulinotropic effects of exendin-4.

### Effect of exendin-4 on islet size

The pancreas of Cre^−^ mice treated with exendin-4 (Cre^−^Ex^+^) had significantly larger-sized islets than controls (134.1 ± 12.2 μm vs 62.9 ± 5.6 μm, p < 0.05) (Fig. 1E). This 2-fold ratio fits well with the increases in β-cell mass and insulin content. There is no effect of exendin-4 in Cre^+^Ex^+^ mice (63.1 ± 5.5 μm, p < 0.05) furthering the hypothesis that EGFR mediates the effects of exendin-4.

### Effect of exendin-4 on glucose-stimulated insulin secretion (GSIS)

At low glucose concentrations (2.8 mmol/L), exendin-4 treated control islets *in vitro* (Cre^−^Ex^+^) have a significantly increased insulin secretion compared to control (Cre^−^Ex^−^) islets, and to Cre^+^ islets with or without exendin-4 treatment. At high glucose concentrations (16.7 mmol/L), exendin-4 treated control islets (Cre^−^Ex^+^) again showed increased insulin secretion. Interestingly, insulin secretion by Cre+ islets is reduced compared to Cre^−^Ex^−^ controls, and is further 2-fold less than controls treated with exendin-4 (Cre^−^Ex^+^). This result suggests a baseline dependence on EGFR to respond to a glucose load. The lack of a rescue effect with exendin-4 treatment implies that the GLP-1 functions through EGFR in this effect.

### Exendin-4-induced β-cell proliferation

**Figure 2:**
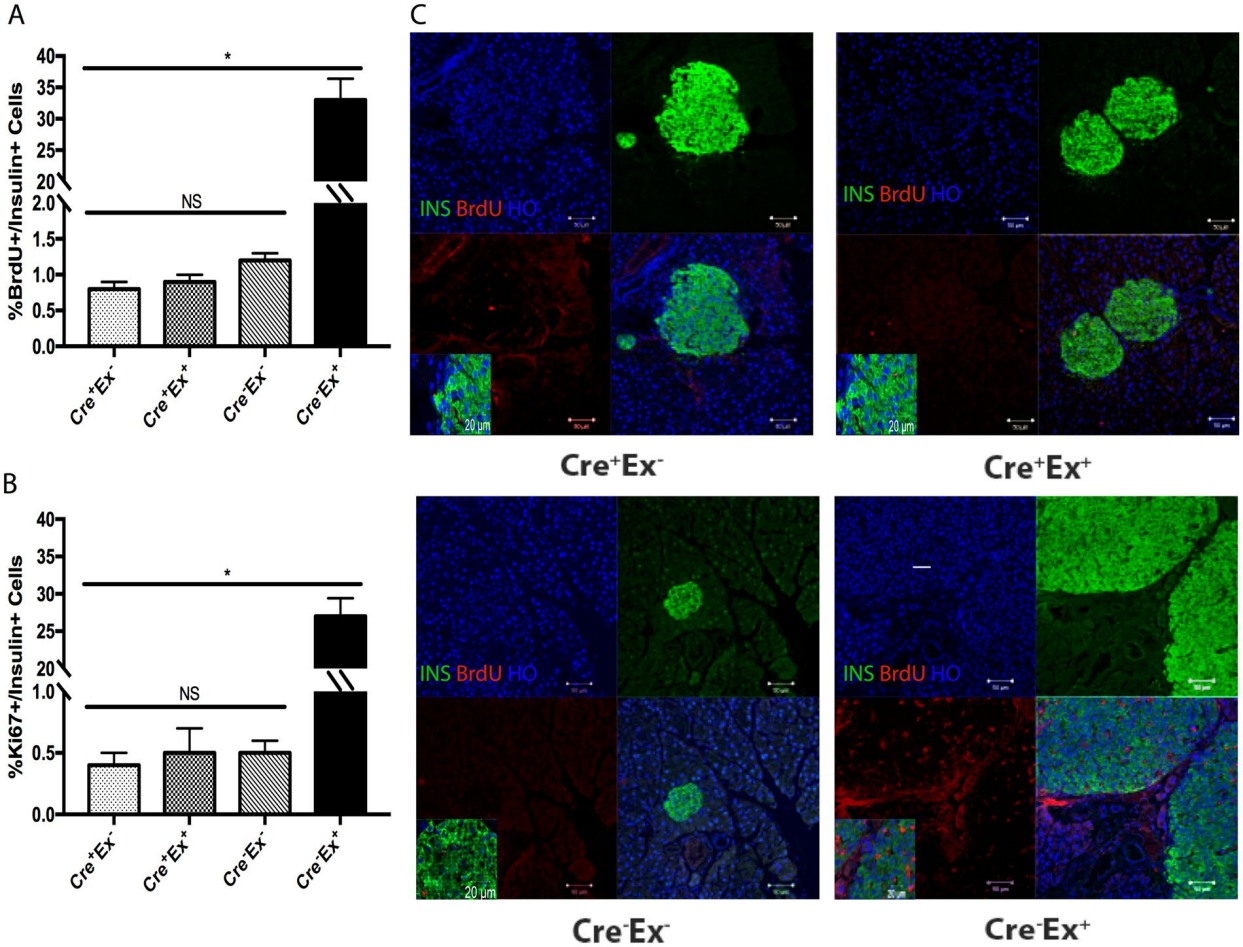
BrdU and Ki67 positive cells within each treatment group. (**A**) There is a 20-fold increase in %BrdU^+^/insulin^+^ cells in control Cre^−^Ex^+^ mice when treated with exendin-4 as compared to untreated Cre^−^Ex^−^ controls. There is no effect of exendin-4 in Cre^+^Ex^+^ mice. (**B**) There is a 20-fold increase in %Ki67^+^/insulin^+^ cell numbers in control mice treated with exendin-4 (Cre^−^Ex^+^) as compared to saline control (Cre^−^Ex^−^). Again, there is no effect of exendin-4 in Cre^+^Ex^+^ mice. (**C**) Representative sections show the large islets in exendin-4 treated control Cre-Ex + mice, with many BrdU^+^ proliferating cells. n = 5. All results were mean ± S.E. *p < 0.05, NS, no significance).

Mice received BrdU in the drinking water for 1 week beginning on day 3 of exendin-4 or saline injection to aid in the analysis of β-cell replication. Ki-67 expression in β-cells from these mice was also analyzed at harvest. BrdU should label all of the proliferating cells during the 7-day exposure to BrdU, while Ki-67 labels cells within the G1 to M phase of the cell cycle at the time the pancreas is harvested^16^. In saline-injected mice, the ratio of BrdU+/Insulin+ cells to all Insulin+ cells at baseline was 1.2 ± 0.1% in control Cre^−^Ex^−^ mice, and 0.8 ± 0.1% in Cre^+^Ex^−^mice (Fig. 2A,C). In exendin-4 treated control mice (Cre^−^Ex^+^), the ratio was strikingly elevated, at 38 ± 3.4%, but remained only 0.9 ± 0.1% in Cre^+^Ex^+^mice. Ki-67 data showed similar results (Fig. 2B).

## Discussion

Reagents that promote β-cell proliferation are potentially very important as treatment modalities for diabetes mellitus. GLP-1 is one hormone signaling pathway useful for therapeutic intervention^21^, although the exact mechanism of action has yet to be completely elucidated. We show that a long acting GLP-1 analogue improves glucose tolerance, increases insulin content, and increases proliferation. Further, by employing a β-cell specific EGFR null mouse, we show that β-cell EGFR is necessary for these effects of GLP-1/exendin-4.

Previous studies have shown that GLP-1 signaling stimulates insulin gene expression and biosynthesis^22^, enhances glucose-stimulated insulin secretion^23^, restores glucose competence in glucose-resistant β-cells^24^, and stimulates β-cell mass expansion^20^. In addition, GLP-1 receptor null mice develop glucose intolerance with dysmorphic islets^25^. GLP-1 clearly acts as a β-cell growth factor in animal models, leading to increased insulin synthesis and β-cell mass. When administered to type 2 diabetics, GLP-1 increases insulin secretion and decreases blood glucose and glucagon^9, 23^.

Here, we confirm a robust improvement in glucose tolerance and insulin content (in direct proportion to β-cell mass) as a result of exendin-4 treatment. The increase in β-cell mass can result from either hypertrophy of existing β-cells or from replication and neogenesis of β-cells. The high percentage of BrdU-incorporated β-cells in exendin-4-treated mice suggests that the increased β-cell mass mainly results from augmentation of β-cell proliferation. The relatively high percentage of Ki67 positive cells that represent cells in an active cell cycle, as compared to BrdU positive cells that represent any proliferation over the 7 days, implies that maximal replication likely occurs at 7 days (time of analysis). This increased β-cell mass correlates with increased insulin content and enhanced glucose tolerance. These effects are limited to the β-cells, as increases in proliferation were not seen in non- β-cell islet cells, nor in acinar nor ductal cells. The absence of proliferation within the acinar or ductal compartments also relates to the current controversy about the potential risk for neoplastic proliferation in patients treated with GLP-1 analogues. Without significant increases in BrdU or Ki67 staining, neoplastic changes in the exocrine compartment would seem unlikely, although endocrine neoplasia would still seem to be a possibility.

GLP-1 is thought to exert its effects by interacting with its high affinity receptor (GLP-1R), which is a member of the G-protein coupled receptor (GPCR) family. The G-protein activation results in the second messenger cascade of cAMP and protein kinase A activation^26^. There is also some evidence that cytosolic calcium may be increased via the cAMP second messenger^27^. The end result is activation of PI3-kinase and its downstream effectors that lead to β-cell proliferation^28^. The precise mechanism of activating the proliferative pathway via the PI3-kinase pathway is not clear. Recent studies suggest that GLP-1R lacks kinase activity, and may rely on EGFR to promote proliferation^29^. Other GPCR agonists have shown reliance upon the EGFR to activate PI3-kinase, including lysophosphatidic acid^29^, thrombin^30^, and angiotensin II^29^.

In this study, exendin-4 seems to fit with the other GPCR agonists in a dependence on EGFR. After 5 consecutive days of tamoxifen injection followed by a 2-week waiting period to allow for degradation of pre-existing EGFR, the majority of the EGFR in the β-cell specific EGFR knockout mice is gone as shown previously^8^. Tamoxifen injection in adult mice to activate the Cre system avoids the effects of EGFR deletion on embryological development that occur with the insulin promoter driving cre, without an ERT, and specifically β-cell development and maturation. Importantly, our previous work also demonstrated that the Pdx1-CreERT line employed here does not contain the human growth hormone mini-gene that could confound the study^8^ nor does the system have leakage into the hypothalamus^8^, as reported in a recent study^31^. In β-cell specific null mutants, the increase in β-cell mass with exendin-4 treatment is lost along with the increases in β-cell proliferation and insulin content. A state of relative glucose intolerance develops when EGFR is deleted from β-cells, suggesting a direct link to insulin secretion and a diminished level of baseline β cell proliferation, consistent with previous work^8^. This result is independent of the proliferative effects of EGFR on β-cell function, which suggests an integral role for EGFR in the coordination of insulin secretion. Although pronounced improvements in β-cell mass and glucose homeostasis are seen with exendin-4 administration in control mice, none of those improvements are seen in the β-cell EGFR null mutants. These data taken together implicate EGFR as a critical pathway from GLP-1 to PI3-kinase and β-cell proliferation. Activation of the EGFR pathway alone may not be sufficient to produce the effects of GLP-1 and exendin-4, but these data suggest that it is necessary. Continued study of the crosstalk among signaling pathways is vital to fully understand β-cell proliferation.

## Electronic supplementary material

Supplemental Figure 1
