# Supplementary material for: GLP-1/Exendin-4 induces β-cell proliferation via the epidermal growth factor receptor
Source: Sci Rep. 2017 Aug 22;7:9100. doi: 10.1038/s41598-017-09898-4 (PMC5567347; doi:10.1038/s41598-017-09898-4)
Supplement: Supplementary file 1 — Supplemental Figure 1 [file 41598_2017_9898_MOESM1_ESM.pdf]

GLP-1/Exendin-4 induces β-cell proliferation via the epidermal growth factor receptor

Joseph Fusco[1], Xiangwei Xiao[1], Krishna Prasadan[1], Qingfeng Sheng[2], Congde Chen[3], Yung-Ching Ming[4], George Gittes[1]*

[1]Children's Hospital of Pittsburgh of the University of Pittsburgh Medical Center, Department of Pediatric Surgery, Pittsburgh, PA 1522

[2]Shanghai Children's Hospital, Shanghai Jiao Tong University, Department of General Surgery, Minhang Qu, 200240, China

[3]The 2nd Affiliated Hospital & Yuying Children's Hospital of Wenzhou Medical University, Wenzhou, Zhejiang, 3250527, China

[4]Chang Gung Memorial Hospital, Chang Gung University, Department of Pediatric Surgery, Taipei City, 105, Taiwan

*** GittesGK@upmc.edu**

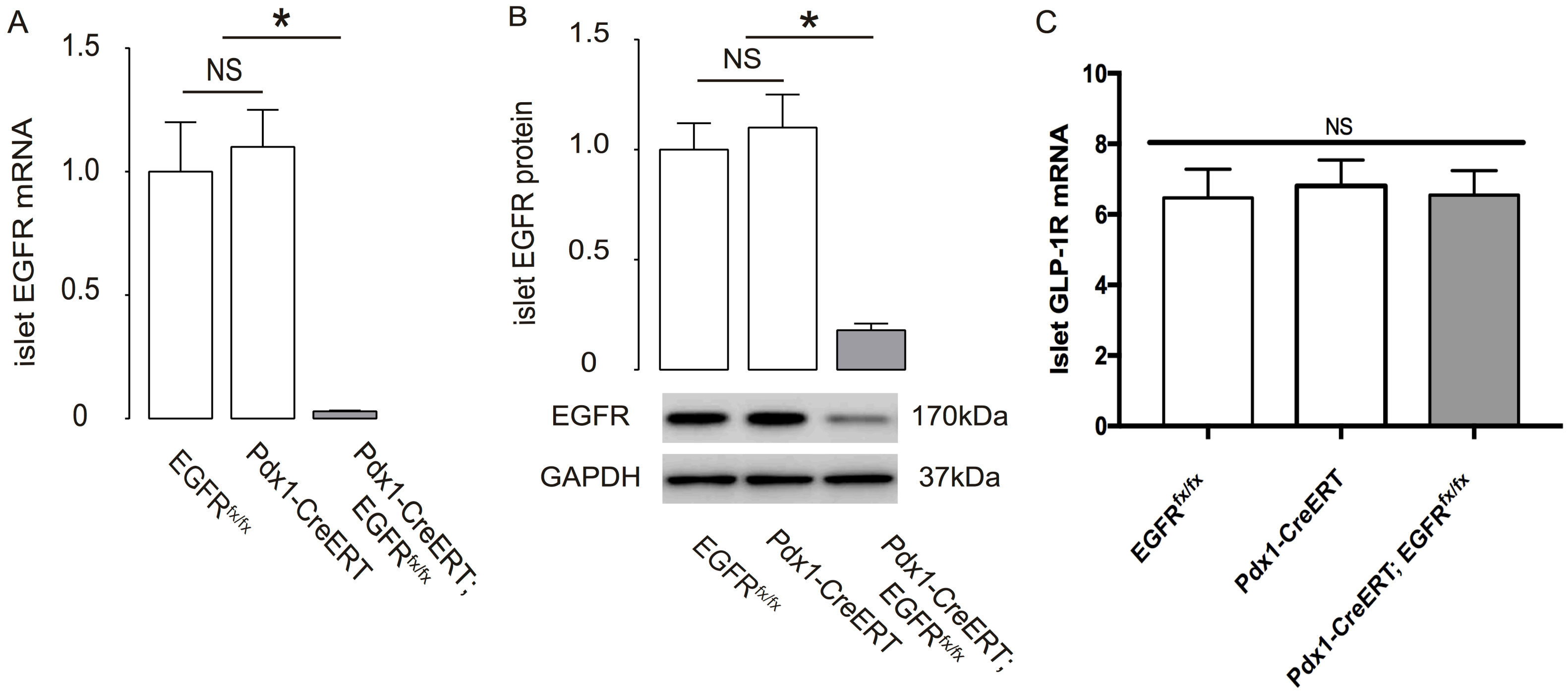

Supplemental Figure 1: (A,B) EGFR levels in mouse islets were determined 2 weeks after tamoxifen injections in Pdx1-CreERT; EGFR$^{fx/fx}$ mice, and control Pdx1-CreERT and control EGFR$^{fx/fx}$ mice, by RT-qPCR (*B*, $p < 0.05$), and by Western blotting analysis (**B**, $p < 0.05$). **(C)** GLP-1R levels in mouse islets were determined 2 weeks after tamoxifen injection in Pdx1-CreERT; EGFR$^{fx/fx}$ mice, and control Pdx1-CreERT and control EGFR$^{fx/fx}$ mice, by RT-qPCR (C, $p > 0.05$).
